# Supplementary material for: Comparative genomics provides new insights into the diversity, physiology, and sexuality of the only industrially exploited tremellomycete: Phaffia rhodozyma
Source: BMC Genomics. 2016 Nov 9;17:901. doi: 10.1186/s12864-016-3244-7 (PMC5103461; doi:10.1186/s12864-016-3244-7)
Supplement: Additional file 6: — List of orphan genes with links to PFAM (related to Additional file 1: Table S1). (ZIP 1428 kb) [file 12864_2016_3244_MOESM6_ESM.zip › PFAM_UNIQUE/G04061_P.html]

#                                                                            --- full sequence --- -------------- this domain -------------   hmm coord   ali coord   env coord
  
# target name        accession   tlen query name           accession   qlen   E-value  score  bias   #  of  c-Evalue  i-Evalue  score  bias  from    to  from    to  from    to  acc description of target
  
#------------------- ---------- ----- -------------------- ---------- ----- --------- ------ ----- --- --- --------- --------- ------ ----- ----- ----- ----- ----- ----- ----- ---- ---------------------
  
G04061\_P             -            235 DUF3727              PF12527.3    100      0.18   10.6   2.3   1   2     0.019        57    2.6   0.0    44    69    13    39     5    72 0.73 - Protein of unknown function (DUF3727)  
G04061\_P             -            235 DUF3727              PF12527.3    100      0.18   10.6   2.3   2   2   0.00071       2.1    7.2   0.5    20    57   107   143   102   151 0.71 - Protein of unknown function (DUF3727)  
G04061\_P             -            235 SERTA                PF06031.8     38     0.038   12.0   0.1   1   2   1.8e-05      0.11   10.5   0.0     4    28    18    44    15    47 0.76 - SERTA motif  
G04061\_P             -            235 SERTA                PF06031.8     38     0.038   12.0   0.1   2   2      0.17     1e+03   -2.2   0.0    28    35    66    73    65    74 0.85 - SERTA motif
